# Towards a neuroscience-based theory of personality: within-subjects dissociation of human brain activity during pursuit and goal conflict

**DOI:** 10.1017/pen.2019.2

**Published:** 2019-07-31

**Authors:** Adam M. Perkins, Rebecca Strawbridge, Danilo Arnone, Steven C. R. Williams, David Gasston, Anthony J. Cleare, Owen O’Daly, Veena Kumari, Ulrich Ettinger, Philip J. Corr

**Affiliations:** 1 Department of Psychological Medicine, Institute of Psychiatry, Psychology & Neuroscience King’s College London, London, UK; 2 Department of Psychiatry, College of Medicine and Health Sciences, United Arab Emirates University, United Arab Emirates; 3 Faculty of Medicine, Universidad Finis Terrae, Santiago, Chile; 4 Department of Neuroimaging, Institute of Psychiatry, Psychology & Neuroscience, King’s College London, London, UK; 5 South London and Maudsley NHS Foundation Trust, London, UK; 6 Centre for Cognitive Neuroscience, Department of Life Sciences, Brunel University London, London, UK; 7 Department of Psychology, University of Bonn, Bonn, Germany; 8 Department of Psychology, City, University of London, London, UK

**Keywords:** threat, anxiety, fear, goal conflict

## Abstract

As demonstrated by neuroimaging data, the human brain contains systems that control responses to threat. The revised Reinforcement Sensitivity Theory of personality predicts that individual differences in the reactivity of these brain systems produce anxiety and fear-related personality traits. Here we discuss some of the challenges in testing this theory and, as an example, present a pilot study that aimed to dissociate brain activity during pursuit by threat and goal conflict. We did this by translating the Mouse Defense Test Battery for human fMRI use. In this version, dubbed the Joystick Operated Runway Task (JORT), we repeatedly exposed 24 participants to pursuit and goal conflict, with and without threat of electric shock. The runway design of JORT allowed the effect of threat distance on brain activation to be evaluated independently of context. Goal conflict plus threat of electric shock caused deactivation in a network of brain areas that included the fusiform and middle temporal gyri, as well as the default mode network core, including medial frontal regions, precuneus and posterior cingulate gyrus, and laterally the inferior parietal and angular gyri. Consistent with earlier research, we also found that imminent threat activated the midbrain and that this effect was significantly stronger during the simple pursuit condition than during goal conflict. Also consistent with earlier research, we found significantly greater hippocampal activation during goal conflict than pursuit by imminent threat. In conclusion, our results contribute knowledge to theories linking anxiety disorders to altered functioning in defensive brain systems and also highlight challenges in this research domain.

Establishing a causal neuroscience-based theory of human personality is a scientific challenge for multiple reasons. One part of the challenge relates to the tendency for early work to be done in rodents, creating the difficulty of extrapolating findings to humans. Nowhere is this clearer than in what could be termed the “experiment-knowledge problem”, which arises because rodents are unaware that they are in an experiment, whereas human volunteers know the situation is artificial. This problem is particularly marked in studies of defensive behaviour: because threat is required for such experiments to be meaningful, yet the informed consent process ensures that human subjects know no harm will come to them – this is quite unlike a naturalistic setting. Such human awareness raises questions about the extent to which findings are comparable to those of rodent studies that entail (perceived) life-and-death situations.

A second part of the challenge relates to differing demand characteristics of experiments seeking to delineate general neural processes versus those seeking to investigate individual differences in the functioning of those processes. The former are best served by powerful stimuli so that behaviour is homogenized, thus providing maximum statistical power. The latter require mild stimuli so that individual differences in responses (heterogeneity) can be detected. This is illustrated by the concept of “defensive distance”, which represents a psychological construct of perceived threat intensity (Blanchard & Blanchard, 1989; Gray & McNaughton, 2000; McNaughton & Corr, 2004). According to this concept, individuals with a short perceived defensive distance respond to distant threats as if they are closer, whereas individuals with a long perceived defensive distance will respond to close threats as if they are more distant (see Table 1, adapted from Corr & Perkins, 2006).

**Table 1.** Relationship between perceived defensive distance and real distance to threat

System state (i.e., personality)	Defensive distance	Real distance sufficient to elicit reaction
High defensive individual	Perceived distance < actual distance	Long
Normal defensive individual	Perceived distance = actual distance	Medium
Low defensive individual	Perceived distance > actual distance	Short

The notion that there exists a continuum of perceived defensive distance is a powerful unifying principle for the neuroscience of personality because there is evidence that neuroticism reflects, in part at least, individual differences along this continuum (Perkins, Arnone, Smallwood, & Mobbs, 2015). Mundane objects or situations that are regarded as innocuous by average individuals will be viewed as threatening by individuals with a short perceived defensive distance, causing them to live their lives in a near-constant state of negative emotionality – that is, to be neurotic. This insight explains why high scores on neuroticism are a risk factor for all forms of psychiatric illness (Claridge & Davis, 2001; De Moor et al., 2015, Kotov, Gamez, Schmidt, & Watson, 2010) and suggests that research which conclusively delineates the neural basis of neuroticism is likely to have considerable clinical significance.

Perceived defensive distance is, however, of little use if it cannot be operationalized in an experimental setting. A practical option in humans is to vary threat distance at a fixed threat intensity via a task resembling a computer game. This option has been pioneered successfully in research showing a shift in neural activity from forebrain (ventromedial prefrontal cortex) to midbrain (periaqueductal gray; PAG) as threat moves closer (Mobbs et al., 2007, 2009, 2010). The Joystick Operated Runway Task (JORT; Figure 1a) has been used to explore this topic from the perspective of individual differences. JORT is a translation of the Mouse Defense Test Battery (MDTB; Griebel, Sanger, & Perrault, 1997), which allows alternating exposure to pursuit and goal conflict. It shows that neuroticism scores correlate positively with flight intensity, suggesting that neuroticism does indeed reflect individual differences in perceived defensive distance (Perkins et al., 2013).

**Figure 1.**
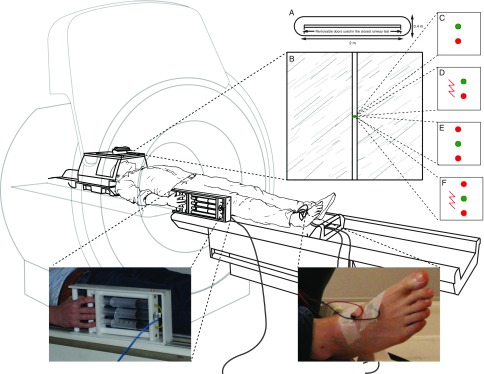
(A) The Mouse Defense Test Battery. (B) The Joystick Operated Runway Task. A force-sensing interface controls the speed of a green dot cursor pursued along a runway by a red dot cursor capable of inflicting electric shock. The task comprised 12 trials each of pursuit (C), pursuit plus threat of electric shock (D), goal conflict (E), goal conflict plus threat of electric shock (F). Illustration by Nick Boon.

A second major concept in the emerging neuroscience of individual differences in human defense is defensive direction, which posits that anxiety is elicited by threats that must be approached (i.e., that generate goal conflict), whereas fear is elicited by threats that need not be approached (i.e., that do not generate goal conflict; Gray & McNaughton, 2000; McNaughton & Corr, 2004). Key to this concept is the septo-hippocampal system which is hypothesized to act as a negatively weighted comparator, typically selecting the least risky option as the correct course of action during goal conflict. Evidence for this concept has been provided by rodent studies, which have guided its development (Gray & McNaughton, 2000).

Testing the defensive direction theory in humans is at an early stage, and evidence so far is mixed. The strongest evidence for the concept of defensive direction has so far come from studies of threat-related facial expressions. In both UK and Brazilian subjects, scenarios featuring threats that need not be approached elicited a facial expression that was recognized by naïve observers as representing fear. In contrast, scenarios featuring ambiguously threatening situations (entailing goal conflict) elicited a risk-assessing facial expression labeled as representing anxiety (Borges, Ferrer-Rosa, Juruena, & Estanislau, 2018; Perkins, Inchley-Mort, Pickering, Corr, & Burgess, 2012).

Data from drug studies with JORT are partially supportive of the defensive direction concept: risk assessment intensity (forwards-backwards oscillation elicited by avoid-avoid goal conflict) was modulated by the anxiolytic drug lorazepam, but only in individuals with low trait anxiety, as if the drug was insufficient to affect the behaviour of high-anxious individuals (Perkins et al., 2009, 2013). Concurrently, in the 2013 study, lorazepam modulated simple avoidance behaviour in interaction with personality. It reduced flight intensity in participants with high fight–flight–freeze (FFF) sensitivity but increased flight intensity in participants with low FFF sensitivity (as measured by the Tissue Damage Fear scale of the Fear Survey Schedule; Wolpe & Lang, 1977).

This apparent conundrum presented by the dual effect of lorazepam might be explained by referring to the aforementioned “experiment-knowledge problem”. In this case, the defensive direction theory dictates that, unlike rodents, human subjects who volunteer to participate in an experiment involving noxious stimuli are exposed to an approach-avoidance goal conflict because they are approaching a threat. This means the experiment is inherently anxiety-generating; hence antianxiety drugs should have a global effect on behaviour throughout the experiment, even in trial types that contain no explicit goal conflict, such as the simple avoidance phase of JORT.

These considerations do not necessarily mean that individual trial types within the human experiment are meaningless; rather, it suggests that a specific trial type containing goal conflict will have less scope to show an anxiety effect if the experiment already is a (albeit implicit) goal conflict. In practice, a smaller distinction might be found in human experiments between activity in the brain systems that govern simple avoidance and goal conflict than is clearly evident in rodent experiments. However, we must be wary of the perils of *post hoc* justifications for predictive failure. This possibility constitutes yet another challenge in neuroscientific models of personality.

In this paper we describe a small-scale pilot study that makes an initial attempt at dissociating neural activity of these systems in humans. We used an fMRI-compatible version of JORT as it allows within-subjects dissociation of neural responses to simple avoidance and goal conflict. Moreover, the task has been proved to be sensitive to drugs with clinical effectiveness for anxiety disorders in both rodent and human versions (Griebel, Perrault, & Sanger, 1998; Perkins et al., 2009, 2013; Stemmelin et al., 2008).

Based on previous research (Mobbs et al., 2007, 2009, 2010), we predicted an increase in the activation of PAG during pursuit by imminent threat, and the anterior hippocampus during goal conflict (Ito & Lee, 2016). We also explored relationships between brain activation and self-reported negative affect to investigate neural processes underpinning individual differences in neuroticism. This is a clinically important issue because high scores on neuroticism increase the risk of psychiatric illness (Claridge & Davis, 2001; De Moor et al., 2015; Kotov, Gamez, Schmidt, & Watson, 2010). As part of this second aim, we predicted that neuroticism scores would modulate hippocampal activity, since rodent data link anxiety to hippocampal activity (Gray & McNaughton, 2000).

## Participants and methods

Twenty-four healthy participants (11 male, 13 female; mean age 25.2, SD ± 5.6) completed the study. All were right-handed, with normal or corrected vision. Participants gave written informed consent, and the study was approved by the King’s College Hospital Research Ethics Committee.

During JORT, participants viewed a computer monitor displaying a runway (Figure 1b). In pursuit trials (Figure 1c), they used a joystick to move a green dot cursor along it, fast enough to stay ahead of a pursuing red dot cursor. The joystick was force-sensing: the greater the force that participants applied to the handle, the faster the green dot cursor moved along the runway. In 50% of pursuit trials, a lightening icon was displayed in the corner of the screen (Figure 1d), signifying that if the red dot cursor touched the green dot cursor, the participant would receive an electric shock (threat condition). JORT delivers electric shocks to the right foot using a custom-built fMRI-compatible electrical stimulator. Each participant chose a shock level they found annoying but not painful, from a choice of eight levels (maximum 80 volts at 20 mA).

Goal conflict trials replicated pursuit trials except that a second red dot traveled ahead of the green dot at a constant velocity (Figure 1e), requiring the participant to move the green dot cursor fast enough to avoid the pursuing red dot cursor, but not so fast that it collided with the leading red dot cursor. In 50% of goal conflict trials, the lightening icon was visible, signifying the participant would receive an electric shock if either of the red dot cursors touched the green dot cursor (Figure 1f).

To enhance unpredictability, trials were presented in a pseudo-random order (i.e., each participant was presented with the same predetermined order of trials that appeared randomly shuffled), and inter-trial intervals were varied pseudo-randomly between 15 and 30 seconds. During inter-trial intervals, the runway remained on screen with the green dot cursor visible: no red dot cursors were visible. Trials terminated if the participant received an electric shock. However, even if a trial terminated early, the next trial did not start early. This prevented participants from accelerating the experiment by trying to get caught quickly. If the participant successfully maneuvered the green dot cursor for 7 seconds, the trial automatically terminated.

The force-sensing joystick apparatus (PH-JS14-MRI; Psyal, London, UK; Figure 1) resembles a grip-strength dynamometer and allows participants to compress three 60-mL plastic syringes using their right hand. The center syringe was sealed and acted as a return spring. The other syringes were connected by plastic tubing to a transducer that generated digital signals from changes in air pressure caused by changes in grip force. The digital signals controlled the speed of the green dot cursor along the runway. The force required to keep the green dot cursor ahead of the red dot cursor was set at 7.5 kg, as pilot testing showed that participants could generate 7.5 kg with one hand for the duration of forty-eight 7-second trials without suffering noticeable fatigue. To further minimize fatigue, participants were instructed to squeeze the joystick handle only when the red dot cursor was on screen – this meant that the participant would be squeezing the handle for a maximum of 336 seconds, spread over the 17-minute duration of the task. Participants practiced with JORT prior to undergoing MRI scanning until they successfully completed each of the four trial types. This ensured that all participants began the MRI session with approximately equal proficiency at operating JORT.

Participants completed the Eysenck Personality Questionnaire–Revised (EPQ-R; Eysenck & Eysenck, 1991) which conceptualizes neuroticism as individual differences in proneness to negative emotions of all kinds (Eysenck, 1967). At the end of the scanning session, participants rated the degree of dread they experienced when the red dot cursor was chasing them, with 1 representing no dread and 10 representing maximum dread.

Blood oxygenation level-dependent (BOLD) fMRI data were acquired on a 3T GE MR750 system (GE Medical Systems, Milwaukee, Wisconsin). T_2_*-weighted gradient echo planar images (EPIs) were acquired every 2 seconds (repetition time) with an isotropic 3 × 3 mm in-plane resolution. The echo time was 30 milliseconds, the flip angle was 75°, and the matrix size was 64 × 64 voxels. Whole-brain coverage was achieved with 39 slices (slice thickness 3 mm, inter-slice gap 0.3 mm); 540 whole-brain volumes were acquired in total. Additionally, a whole-brain high-resolution sagittal Magnetization Prepared Rapid Acquisition GRE 3D Inversion Recovery (MP-RAGE) structural scan was acquired with an inversion time of 400 milliseconds, an echo time of 3.016 milliseconds, repetition time of 7.312 seconds, and a flip angle of 11°. The volume consisted of 196 contiguous slices with a slice thickness of 1.2 mm. Data quality was assured using an automated quality control procedure (Simmons, Moore, & Williams, 1999).

EPI data were pre-processed with version 8 of Statistical Parametric Mapping package (SPM8, revision 4667; http://www.fil.ion.ucl.ac.uk/spm/software/spm8/) running in MATLAB 7.2.0 (The MathWorks, Inc., 2006). The fMRI time series was first corrected for slice timing differences, then realigned to correct for volume-to-volume head motion with a cut-off of 3 mm (i.e., one voxel). The image time series was normalized to template space via unified segmentation of the MP-RAGE structural image. Finally, the normalized data were spatially smoothed using an 8-mm full-width at half maximum kernel.

Data were analyzed under the SPM8 general linear model. For each participant, a separate fixed-effects analysis was conducted and involved building a model with four regressors encoding onset and offset of 12 trials for each JORT condition (i.e., pursuit trials without threat of potential shock; pursuit trials with threat of potential shock; conflict trials without threat of potential shock; and finally conflict trials with the threat of potential shock). The resultant box-car time series was convolved with the canonical hemodynamic response function. Additionally, a parametric modulator encoding peak threat (i.e., the peak value of the inverse distance from the pursuer) was added for each condition, resulting in a total of eight regressors. Six regressors encoding the volume-to-volume motion (translations and rotations) were also included in the model as regressors of no interest to control for signal intensity variation resulting from participant head movement.

The primary contrasts were the task conditions compared to the implicit baseline-resting condition. In addition to task activation, contrasts for parametric modulators were created. Following parameter estimation, these contrasts of beta-coefficients were taken forward to group-level random-effects analyses. These models included: (i) a 2 × 2 repeated-measures factorial ANOVA including four contrast images per participant (e.g., four activation contrasts, or contrasts for each of the peak threat parametric modulators); and (ii) regression models exploring the relationship between individual differences response (i.e., beta-coefficients) in a given condition and post-scan ratings of subjective dread during exposure to the red dot cursor as well as questionnaire data on trait individual differences in proneness to negative emotions (i.e., EPQ neuroticism scores). Age was included as a nuisance covariate in all group-level models.

As this is the first fMRI JORT study, we characterized task-related activations and deactivations, then the main effects of Threat versus No Threat, and separately Pursuit versus Conflict. Finally, we tested for brain regions where activity is influenced by an interaction of these two factors (i.e., threat of electric shock and number of pursuers). For these exploratory whole-brain analyses, Gaussian random field theory was employed for determining thresholds for multiple-comparison correction. Significance was defined as results surviving family-wise error (FWE) correction for multiple comparisons based on cluster extent (*p* < 0.05) given a default cluster-forming threshold of *p* < 0.001.

Our *a priori* interests were in the sensitivity of PAG to pursing threat and conflict sensitivity of the anterior hippocampi. Regions of interest (ROIs) defining these structures were generated independently of the data to avoid circularity (Kriegeskorte, Simmons, Bellgowan, & Baker, 2009). The hippocampal ROIs were bilateral anatomical masks generated from the automatic anatomical labeling atlas with the WFU-Pickatlas toolbox (Wake Forest University). As we are not aware of anatomically defined masks for PAG, our ROI for this brainstem region was defined as a sphere with a 6-mm-radius centering on the PAG peak previously linked to the processing of threat proximity (*x* = 4, *y* = −30, *z* = −24), using the Montreal Neurological Institute space (Mobbs et al., 2010). These hypotheses were tested using small-volume correction with significance defined as results surviving peak-level FWE correction (*p* < 0.05). The same ROI was also used to extract mean threat-related BOLD signal from PAG for subsequent regression analyses.

## Results

Table 2 presents descriptive statistics and correlations between behavioural criteria, self-reported negative affect, and number of electric shocks received by the participants. Neuroticism scores were significantly negatively correlated with the number of electric shocks received, and dread ratings were significantly positively correlated with the number of electric shocks. Female participants, on average, chose a significantly lower level of electric shock compared with males and, unlike males, displayed a significant negative correlation between neuroticism and the level of electric shock selected. There were no significant positive correlations between affective measures and the intensity of either form of threat avoidance behaviour.

**Table 2.** Descriptive statistics and inter-correlations for self-reported negative affect and behavioural criteria

Variable	Overall mean (SD)	Male mean (SD)	Female mean (SD)	1	2	3	4	5	6
1. Neuroticism	8.92 (6.87)	9.09 (6.93)	8.77 (7.07)	−	−.017	.166	.258	−.045	−.442
					−.702**	.293	.114	−.164	−.590*
2. Level of electric shock	4.63 (1.24)**	5.45 (1.21)	3.92 (0.76)	−.222	−	−.456	.131	−.026	−.286
						−.277	−.222	−.006	.564*
3. Dread ratings	4.29 (2.35)	3.82 (2.18)	4.69 (2.50)	.231	−.392	−	.365	.599	.694*
							−.024	−.117	.310
4. Flight intensity	−0.12 (0.87)	−0.37 (1.11)	0.10 (0.57)	.178	−.144	.231	−	.797**	.103
								.251	−.479
5. Risk assessment intensity	−0.02 (0.12)	−0.05 (0.16)	−0.00 (0.08)	−.087	−.128	.311	.685**	−	.375
									.247
6. Number of electric shocks	2.17 (2.24)	1.36 (1.96)	2.85 (2.30)	−.505*	−.133	.486*	−.026	.334	−

*Note*. Correlations for the whole sample (*n* = 24) in the lower left half of the matrix; correlations for males (*n* = 11, upper) and females (*n* = 13, lower) in the upper right half of the matrix.

*p < 0.05; **p < 0.01.

### Main effects

A main effect of Condition (i.e., conflict > pursuit) occurred in a large bilateral network extending into the frontal and parietal, premotor and supplementary motor areas, the cerebellum, and insula bilaterally (Table 3). The main effect of Condition (pursuit > conflict) was also present as significantly greater activation in the superior and middle frontal gyri and the inferior parietal lobule extending into the angular gyrus. We found no main effect of the threat of receiving an electric shock.

**Table 3.** Main effects and Condition × Threat interactions

Brain region	Side	Cluster size	*z*-score	MNI coordinates		
				X	Y	X
Pursuit > Conflict						
**Superior frontal gyrus**	**Left**	**2138**	**6.14**	−**12**	**38**	**54**
Superior frontal gyrus	Left		5.36	−22	24	58
Middle frontal gyrus	Left		5.19	−42	12	52
**Angular gyrus**	**Left**	**637**	**5.33**	−**40**	−**70**	**44**
Inferior parietal lobule	Left		4.94	−50	−60	44
Superior temporal gyrus	Left		4.94	−40	−58	26
Conflict > Pursuit						
**Paracentral gyrus**	**Left**	**24266**	**7.83**	−**18**	−**10**	**66**
Superior frontal gyrus	Right		7.8	26	−2	58
Supplementary motor area	Left		6.81	−2	−10	64
Cerebellar vermis (8)	**Right**	**7855**	**7.53**	**4**	−**72**	−**36**
Cerebellar vermis (6)	Right		5.86	26	−54	−26
Anterior cerebellar lobe	Right		5.68	6	−58	−24
**Insula**	**Right**	**1196**	**6.28**	**38**	**20**	−**4**
**Insula**	**Left**	**713**	**4.94**	−**32**	**18**	−**8**
Condition × Threat interaction (deactivation occurred in the trials containing goal conflict plus threat of electric shock, except in the inferior occipital gyrus which showed increased activation during the trials containing goal conflict)						
**Medial prefrontal gyrus**	**Left**	**2785**	**5.25**	−**10**	**66**	**16**
Superior medial frontal gyrus	Medial		4.73	0	66	8
Middle frontal gyrus	Left		4.49	−28	60	12
**Inferior occipital gyrus**	**Left**	**6304**	**4.88**	−**42**	−**78**	−**6**
Fusiform gyrus	Left		4.5	−40	−62	−14
Middle occipital gyrus	Right		4.39	36	−84	0
**Angular gyrus**	**Left**	**1275**	**4.54**	−**48**	−**54**	**28**
Inferior parietal lobule	Left		4.29	−50	−58	40
Middle temporal gyrus	Left		4.13	−34	−66	26
**Frontal pole**	**Right**	**399**	**4.16**	**30**	**50**	**0**
Anterior cingulate gyrus	Right		3.91	20	42	10
Frontal pole	Right		3.46	26	64	6
**Precuneus**	**Medial**	**934**	**3.89**	**0**	−**56**	**26**
Posterior cingulate gyrus	Left		3.73	−2	−40	26
Precuneus	Right		3.89	2	−60	34

*Note.* Cluster information for main effects and task Condition × Threat interactions. All regions survive whole-brain family-wise error correction on the basis of cluster extent (*p*FWE < 0.05) with a default cluster-forming threshold of *p* < 0.001. Main cluster peaks are shown in bold. MNI = Montreal Neurological Institute.

### Interaction

**Figure 2.**
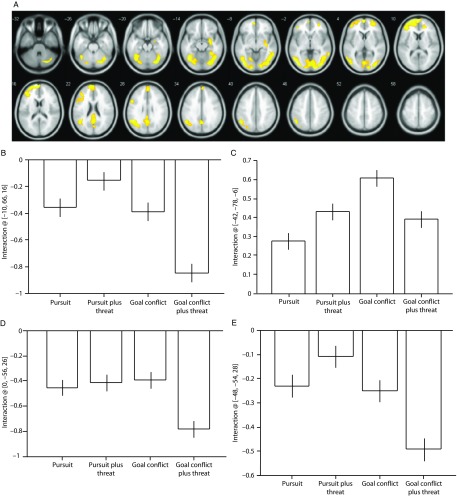
(A) Statistical parametric maps illustrating blood oxygen level-dependent (BOLD) responses for the Condition × Threat interaction. (B) Parameter estimates for activity in the medial prefrontal gyrus [−10, 66, 16]; (C) inferior occipital gyrus [−42, −78, −6]; (D) angular gyrus [0, −56, 26]; (E) precuneus [−48, −54, 28]. All regions survive whole-brain family-wise error correction on the basis of cluster extent (*p*FWE < 0.05) with a default cluster-forming threshold of *p* < 0.001. Error bars represent 1 SEM.

A significant Condition × Threat interaction was observed in the fusiform and middle temporal gyri (Table 3) and brain regions typically characterized as the default mode network (i.e., medial frontal regions, precuneus and posterior cingulate gyrus, and laterally the inferior parietal and angular gyri). The main cluster peaks occurred in the medial prefrontal gyrus (B), the inferior occipital gyrus (C), the angular gyrus (D), and the precuneus (E; Figure 2). The effects at these sites were driven by increased deactivation during the trials containing goal conflict plus threat of electric shock, except for the effects in the inferior occipital gyrus which were driven primarily by increased activation during the trials containing goal conflict.

### Hypothesis testing: hippocampal activation by goal conflict

**Figure 3.**
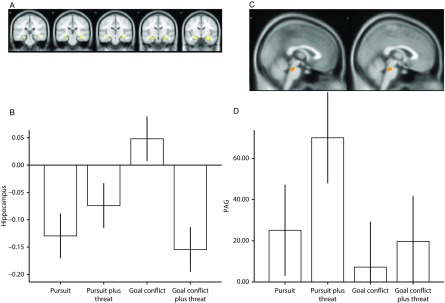
(A) Statistical parametric map illustrating blood oxygen level-dependent (BOLD) responses in the anterior hippocampi during the goal conflict condition (shown at an uncorrected voxel threshold of *p* < 0.005 for display purposes). (B) Parameter estimates for activity in the right anterior hippocampus [32, −14, −12] during the four task conditions. (C) BOLD responses in the periaqueductal gray (PAG) at the point of peak threat proximity during pursuit plus threat of electric shock (shown at an uncorrected voxel threshold *p* < 0.01 for display purposes). (D) Mean BOLD activity in PAG for the task conditions [6 −28 −28]. Error bars represent 1 SEM; the *y*-axis displays beta values that reflect scaling factors for the peak threat value to fit the residual after fitting the mean.

There was a significant, small-volume-corrected interaction (see Figure 3a, 3b) in the right (*p* = 0.008, *z*-score = 4.14; 105 voxels [32, −14, −12]) and left (*p* = 0.045, *z*-score = 3.52; 25 voxels [−24, −8, −18]) anterior hippocampi. This indicated significant deactivation in the anterior hippocampi during all trial types relative to implicit baseline-resting condition, except during trials containing goal conflict, when there was significant activation. Similar patterns occurred in the midbrain (encompassing several nuclei including PAG; *p* = 0.011, *z*-score = 3.35 [8, −30, −32]) and, although non-hypothesized, such patterns of interaction were also evident within the right (*p* = 0.003, *z*-score = 4.05; 67 voxels [30, −8, −12]) and left (*p* = 0.016, *z*-score = 3.47; 24 voxels [−24, −6, −18]) amygdalae.

### Hypothesis testing: trial-to-trial threat proximity and the midbrain

In order to explore the effect on brain activity of trial-to-trial differences in threat proximity (i.e., the inverse of the minimal distance between the green dot cursor and red dot cursor or cursors in each trial), we examined this separately for pursuit plus threat of electric shock and goal conflict plus threat of electric shock. We found that BOLD signal in a midbrain location encompassing several nuclei, including PAG (Figure 3c, 3d), varied on a trial-by-trial basis with the measure of threat proximity (i.e., trial peak threat level) during pursuit plus threat of electric shock (*p* < 0.01, *z*-score = 3.31; 69 voxels [6, −28, −28]): activation was the highest when the threat was closest during this trial type. Follow-up testing found that the same region showed a trend towards significantly greater threat proximity-related responses during the task condition containing pursuit plus threat of electric shock, compared to pursuit without threat of electric shock (*p* = 0.10, *z*-score = 2.09; 14 voxels [6, −28, −28]).

### Regression analysis

**Figure 4.**
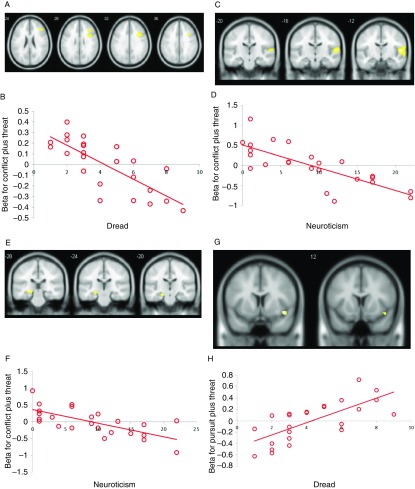
Scatterplots showing effect on BOLD signal of inter-individual differences in dread elicited by threat of electric shock and also neuroticism (as measured by the Eysenck Personality Questionnaire). (A, B) Right dorsolateral prefrontal cortex [32, 12, 34]. (C, D) Right superior temporal gyrus [52, −12, 8]. (E, F) Left hippocampus [−22, −22, −15]. (G, H) Operculum/right posterior insula [48, 8, −12]. Each point represents an individual’s response on the self-report measures.

We explored the relationship between task-related BOLD responses during goal conflict trials plus threat of electric shock, individual differences in state dread, and EPQ neuroticism (Table 4). Dread scores were significantly negatively correlated with BOLD responses in the right inferior operculum and middle frontal gyrus [32, 12, 34] (dorsolateral prefrontal cortex [DLPFC]; Figure 4a, 4b). EPQ neuroticism scores correlated negatively with BOLD responses in the superior and middle temporal gyri [52, −12, 8] (Figure 4c, 4d). A hypothesis-driven ROI analysis showed EPQ neuroticism scores negatively correlated with BOLD activity in the left hippocampus (*p* = 0.019, *z*-score = 3.74; 50 voxels [−22, −22, −16]) during conflict trials under threat of shock (Figure 4e, 4f). Dread scores were significantly positively correlated with task-related activity in a cluster in the operculum extending to the insula [48, 8, −12] pursuit trials under threat (whole brain; Figure 4g, 4h).

**Table 4.** Regression analyses

Region label	Laterality	Cluster size	z-Score	MNI coordinates		
				X	Y	X
Dread vs. Conflict (w. Threat)						
DLPFC*	**Right**	**373**	**4.42**	**32**	**12**	34
Middle frontal gyrus	Right		3.94	38	6	30
Middle frontal gyrus	Right		3.65	42	32	26
EPQ vs. Conflict (w. Threat)						
**Superior temporal gyrus**	**Right**	**457**	**4.42**	**52**	−**12**	**8**
Middle temporal gyrus	Right	3.67	62	−8	−6	

*Note*. Cluster information for brain regions where conflict-related blood oxygenation level-dependent activity, under the threat of shock, was significantly correlated with subjective dread scores and Eysenck Personality Questionnaire scores. All regions survive whole-brain family-wise error correction on the basis of cluster extent (*p*FWE < 0.05) with a default cluster-forming threshold of *p* < 0.001. Main cluster peaks are shown in bold. MNI = Montreal Neurological Institute.

Finally, we assessed the degree to which dread scores associated with midbrain threat sensitivity (i.e., the degree to which trial-to-trial variation in midbrain activity tracked with trial-to-trial variation in peak threat [i.e., threat proximity]). We found a significant main effect of condition (i.e., pursuit > conflict) by covariate (i.e., dread) interaction, *F*(1,20) = 8.549, *p* = 0.008. *Post hoc* one-tailed correlational analyses demonstrated that when controlling for age, PAG threat sensitivity was weakly correlated (partial correlation coefficient = 0.325, *p* = 0.065), but the ANCOVA effect was predominantly driven by the relationship between dread scores and midbrain threat sensitivity during pursuit under threat (partial correlation coefficient = 0.441, *p* = 0.018) with a non-significant effect in pursuit trials without threat of electric shock (partial correlation coefficient = 0.266, *p* = 0.110).

## Discussion

We used JORT to attempt a within-subjects dissociation of brain activity in healthy humans during pursuit and goal conflict. Our over-arching aim in this study was to provide a neuroimaging test of the revised Reinforcement Sensitivity Theory’s principles of defensive distance and direction (Gray & McNaughton, 2000; McNaughton & Corr, 2004). We also sought to begin exploring whether the “experiment-knowledge problem” is a major issue in a study of this type. This refers to the knowledge of human subjects that they are approaching threat by volunteering themselves to receive foot-shocks. This situation sets up a goal conflict that risks generating intrinsic anxiety which in turn may reduce the visibility of any task-specific anxiety induction.

Congruent with the notion that high scores on neuroticism reflect a magnified perception of threat (Gray & McNaughton, 2000), we found a significant negative correlation between neuroticism scores and the number of electric shocks received: it seems the higher participants scored on neuroticism, the more motivated they are to avoid threat. We also found a significant positive correlation between the number of electric shocks received and dread ratings, suggesting that experiencing electric shocks was unpleasant.

In addition to brain activation produced by visual and motor demands of the task, there was a significant main effect of condition, suggesting that the pursuit and goal conflict JORT conditions were sufficiently different to activate the brain differently.

There was no significant main effect of threat, most likely because participants’ neuroticism scores on average were approximately one standard deviation below normal (Perkins, Cooper, Abdelall, Smillie, & Corr, 2010). Had we been able to test a full range of scorers on neuroticism, we might have found threat of electric shock causing a main effect on brain activation – this range-restriction factor means that any interpretations of neuroticism findings in the present study must be regarded as tentative. Another factor may have been that participants chose the intensity of electric shocks so they might have been unpleasant but perhaps not threatening. In an earlier study (Kumari, Das, Wilson, Goswami, & Sharma, 2007), participants were told that the shock they would receive while in the scanner would be of the same intensity they chose or stronger (but did not deliver them). However, we did find a condition × threat interaction: the default mode network (DMN; Raichle et al., 2001) deactivated during goal conflict plus threat of electric shock. Since these two attributes make this trial type the most demanding in attentional terms, this finding fits the notion that DMN is most active when the environment is unstimulating and least active when the environment demands focused attention.

We had two hypothesized results: first, we found a significant positive association between threat proximity and activity in a part of the midbrain encompassing several nuclei, including PAG. Activity peaked when the threat stimulus was at its closest point (Figure 3a). The effect was significantly stronger during pursuit plus threat of electric shock, relative to the other three task conditions (Figure 3b) and resembles findings from a maze-based threat-avoidance task (Mobbs et al., 2007, 2009). Second, activity in the anterior hippocampus increased during goal conflict, relative to the other three task conditions (Figure 3b, 3c). This result resembles that found with a foraging task (Bach et al., 2014).

As JORT allowed us to compare brain activation across all four task conditions within subjects, our most important result is that we did not find significant PAG activity during goal conflict, nor significant activity in the anterior hippocampus during pursuit plus threat of electric shock. This pattern of results, therefore, is consistent with a double-dissociation that supports separate findings by other researchers of midbrain activation in pursuit by threat and anterior hippocampal activation during goal conflict.

It is unclear why the anterior hippocampal circuits of our participants deactivated during the task condition containing goal conflict plus threat of electric shock, relative to the task condition containing only goal conflict (Figure 3b). This condition is the most intense form of goal conflict in JORT and, we anticipated, would cause greater hippocampal activation than during goal conflict without threat of electric shock. One explanation is that during severe threat, the neural circuits governing responses to goal conflict (Behavioral Inhibition System [BIS]; Gray, 1976) deactivate to allow an explosive, panic-based avoidance response. Since the hippocampus is a major component of BIS (Gray & McNaughton, 2000) and the task condition containing goal conflict plus threat of electric shock included two threat stimuli, it is plausible that the threat level in this trial type was sufficiently severe to cause anterior hippocampal deactivation. This is an issue that may have been compounded by the intrinsic anxiety caused by the experimental situation (the experiment-knowledge problem), since it would have meant that participants were already experiencing hippocampal activation, even before beginning the experimental session.

This explanation is supported by our additional finding of a significant negative association between neuroticism scores and hippocampal activation during goal conflict plus threat of electric shock (Figure 4e, 4f). Since neuroticism reflects sensitivity to threat (Perkins et al., 2013), hippocampal activation should correlate negatively with neuroticism, as indeed we found. However, as a caveat, the restriction of range of neuroticism scores in this sample means that our interpretations are tentative.

Based on this analysis and with that caveat in mind, we suggest that during goal conflict without threat of electric shock, low and high scorers on neuroticism both process goal conflict normally, hence the observed tendency for greater hippocampal activation during this task condition. But in a situation containing threat of electric shock in addition to goal conflict, relatively threat-sensitive participants (i.e., higher scorers on neuroticism) experience hippocampal deactivation, whereas the less threat-sensitive participants do not and thus can process goal conflict as normal.

The notion that neuroticism is negatively related to the retention of cognitive control during threat-related goal conflict conditions is backed up by two of our exploratory results – first, the negative association between dread scores and activation in DLPFC during the task condition containing goal conflict plus threat of electric shock (Figure 4a, 4b); second, the finding that the distance effect on the midbrain was the strongest in participants who reported elevated levels of dread when pursued by threat. Since threat-generated increases in negative state affect were associated with deactivation in DLPFC (a component of the cognitive control network; Niendam et al., 2012) but with activation in a midbrain area that is associated with primitive, panic-related responses to threat, these findings back up previous evidence for top-down inhibitory processes upon reactions to threat (Mobbs et al., 2007, 2015).

More specifically, since DLPFC influences executive functioning and PAG panic, it follows that low scorers on neuroticism, since these do not magnify threat, will have a higher threshold for preserving activation in DLPFC during threat-intense goal conflict and thus avoiding panic. This begs the question of where these individual differences in perceptions of threat intensity come from: one candidate is the basolateral amygdala (BLA; Mobbs et al., 2007, 2009), suggesting that individuals who happen to possess particularly reactive BLA circuits will display high scores on neuroticism (Perkins et al., 2015). This may explain why low scorers on neuroticism perform better in roles that require efficient decision-making under threat (e.g., bomb-disposal officer or fighter pilot; Bartram & Dale, 1982; Hallam & Rachman, 1980) than individuals with high scores on neuroticism, who will tend to suffer early hippocampal and DLPFC deactivation and thus have a lower threshold for panic.

Our finding of a negative relationship between EPQ neuroticism scores and BOLD responses in the superior and middle temporal gyri (Figure 4c, 4d) is consistent with previous research (Kumari et al., 2007) which found, using an electric shock task, that neuroticism correlated negatively with brain activity in the superior and middle temporal gyrus, extending to the hippocampus, precuneus, putamen, thalamus, and middle occipital gyrus. Our finding that dread scores were significantly positively correlated with task-related activity in a cluster extending from the operculum to the posterior insula (Figure 4g, 4h) echoes previous research (Coen et al., 2011) that found a positive correlation during the anticipation of visceral pain between neuroticism scores and activity in the insula.

In conclusion, our results provide a small-scale pilot illustration of the challenges that face researchers seeking to explore the associations in healthy humans between midbrain and hippocampal activation and flight from a pursuing threat and goal conflict, respectively. Our most important finding was that, consistent with earlier research, imminent threat activated the midbrain and that this effect was significantly stronger during the simple pursuit condition than during goal conflict. Our results also suggest future studies should pay closer attention to examining and comparing fMRI results in men and women. The complex results regarding hippocampal activation by goal conflict show the limitations of this approach but nevertheless suggest that ethoexperimental computer tasks are useful for understanding human brain activation during risky or threatening situations. Finally, our results contribute information to theories linking anxiety disorders to altered functioning in defensive brain systems.
